# Phase 2 study of ibrutinib plus venetoclax in Japanese patients with relapsed/refractory mantle cell lymphoma

**DOI:** 10.1007/s10147-023-02443-6

**Published:** 2023-12-29

**Authors:** Hideki Goto, Satoshi Ito, Masahiro Kizaki, Masaki Yamaguchi, Noriko Fukuhara, Koji Kato, Toko Saito, Yasuhito Terui, Sumiko Okubo, Tomomi Soshin, Jiewei Zeng, Hideyuki Honda, Mohamed Badawi, Jeremy A. Ross, Koji Izutsu

**Affiliations:** 1 Department of Hematology, Hokkaido University Hospital, Kita-14, Nishi-5, Kita-Ku, Sapporo-Shi, Hokkaido, Japan; 2 Department of Neurology, Hematology, Metabolism, Endocrinology and Diabetology (3rd Department of Internal Medicine), Yamagata University Hospital, 2-2-2 Iida-Nishi, Yamagata-Shi, Yamagata, Japan; 3 Department of Hematology, Saitama Medical Center, Saitama Medical University, 1981 Kamoda, Kawagoe-Shi, Saitama, Japan; 4 Department of Hematology, Ishikawa Prefectural Central Hospital, 2-1 Kuratsuki Higashi, Kanazawa-Shi, Ishikawa, Japan; 5 Department of Hematology, Tohoku University Hospital, 1-1 Seiryo-Machi, Aoba-Ku, Sendai-Shi, Miyagi, Japan; 6 Department of Medicine and Biosystemic Science, Kyushu University Graduate School of Medical Sciences, 3-1-1 Maidashi, Higashi-Ku, Fukuoka-Shi, Fukuoka, Japan; 7 Department of Hematology and Cell Therapy, Aichi Cancer Center Hospital, 1-1 Kanokoden, Chikusa-Ku, Nagoya-Shi, Aichi, Japan; 8 Department of Hematology, Saitama Medical University Hospital, 38 Morohongo, Moroyama-Machi, Iruma-Gun, Saitama, Japan; 9 Japan Development, AbbVie GK, 3-1-21Minato-Ku, Shibaura, Tokyo, Japan; 10 Department of Data and Statistical Sciences, AbbVie, Inc., 1400 Sheridan Rd, North Chicago, IL USA; 11 Department of Clinical Pharmacology, AbbVie, Inc., 1400 Sheridan Rd, North Chicago, IL USA; 12 Department of Precision Medicine, AbbVie, Inc., 1400 Sheridan Rd, North Chicago, IL USA; 13 Department of Hematology, National Cancer Center Hospital, 5-1-1 Tsukiji, Chuo-Ku, Tokyo, Japan

**Keywords:** Venetoclax, Ibrutinib, Mantle cell lymphoma

## Abstract

**Background:**

Despite high response rates to initial therapy, most patients with mantle cell lymphoma (MCL) experience relapsed or refractory (R/R) disease. Here, we report the efficacy, safety, and pharmacokinetics of the Phase 2, single-arm M20-075 study (NCT04477486) of ibrutinib and venetoclax combination therapy in Japanese patients with R/R MCL.

**Methods:**

Patients received 560 mg ibrutinib and 400 mg venetoclax (after a 5-week ramp-up from 20 mg) once daily for up to 104 weeks. Primary endpoint was complete response (CR) rate by independent review committee (IRC). Secondary endpoints included overall response rate (ORR), duration of response (DOR), undetectable minimal residual disease (uMRD) rate, progression-free survival (PFS), overall survival (OS), safety including dose-limiting toxicity (DLT) assessment in the first six patients, and pharmacokinetic parameters. Full analysis set (FAS) comprised all treated patients. Per protocol set (PPS) excluded treated patients with non-evaluable disease at baseline by IRC.

**Results:**

Thirteen patients were treated (FAS *n* = 13; PPS, *n* = 12). Median age was 71 years, patients had a median of two prior treatments. After a median follow-up of 9.6 months, IRC-assessed CR rate and ORR were both 83% (PPS). All six MRD-evaluable patients had uMRD. Median DOR, PFS, and OS were unreached. The most common Grade ≥ 3 treatment-emergent adverse event (TEAE) was neutropenia (23%); 1 patient discontinued due to squamous cell carcinoma of the lung. No DLTs, tumor lysis syndrome, or deaths related to TEAEs were observed.

**Conclusion:**

Ibrutinib plus venetoclax exhibited high response rates and a well-tolerated safety profile in Japanese patients with R/R MCL.

**Supplementary Information:**

The online version contains supplementary material available at 10.1007/s10147-023-02443-6.

## Introduction

Mantle cell lymphoma (MCL) represents approximately 3% of all malignant lymphoma cases in Japan and occurs more commonly in elderly men [1, 2]. Unfortunately, there is no cure for MCL, and nearly all patients inevitably relapse [3, 4]. Furthermore, patients with relapsed/refractory (R/R) MCL have a poor prognosis, with a median overall survival (OS) of 3 to 4 years [5]. Recent advances have presented a shift in standard of care from chemotherapy to targeted therapies for patients with R/R MCL, with Bruton tyrosine kinase inhibitor (BTKi) therapy representing a major therapeutic class for this indication [6]. In the randomized, open-label Phase 3 RAY study in patients with R/R MCL, the first-in-class BTKi ibrutinib exhibited superior efficacy compared to temsirolimus and a more favorable safety profile [7]; these results were substantiated in a 3-year follow up [8]. In a Phase 2 study of ibrutinib monotherapy in Japanese patients with R/R MCL, the ORR was 87.5%, and the complete response (CR) rate was 12.5% for the primary analysis, with a median treatment duration of 6.5 months; the overall response rate (ORR) was 93.8% and the CR rate was 31.3% for the final analysis, with a median follow-up of 22.5 months [9, 10]. Another targeted agent under investigation for the treatment of R/R MCL is the selective B-cell lymphoma 2 (BCL-2) inhibitor venetoclax. In the Phase 1 M12-175 study, an ORR of 75% and a CR rate of 21% was observed with venetoclax monotherapy in patients with MCL, and treatment was generally well-tolerated [11].

Prior preclinical investigations in MCL models have reported synergistic anti-tumor activity with ibrutinib and venetoclax combination [12]. Data from studies evaluating this combination in patients with R/R MCL demonstrated a safety profile that is well-tolerated and high rates of response in patients with both high and low risk of tumor lysis syndrome (TLS). In the AIM study, patients were treated with 560 mg ibrutinib per day and 400 mg venetoclax per day after a ramp-up period initially starting at 50 mg per day; after two patients experienced TLS, the starting dose of venetoclax was amended from 50 mg per day to 20 mg per day, and no subsequent cases of TLS were reported [13]. A CR rate of 71% was observed, and, of the patients with a response, 78% were estimated to have an ongoing response at 15 months. Common adverse events were generally low grade and included gastrointestinal (GI) events. The safety run-in of the Phase 3 SYMPATICO study further investigated the combination of ibrutinib and venetoclax [14]. Patients received concurrent 560 mg ibrutinib and venetoclax starting from 20 mg once daily, ramping up to a target dose of 400 mg venetoclax. There were no occurrences of clinical TLS, and DLTs occurred in 14% of patients [14]. The ORR was 81%, with 62% of patients achieving a complete response [14]. These results served as the basis for further investigation of ibrutinib and venetoclax combination therapy. Here, we report the results from the Phase 2 M20-075 study, which was designed to evaluate the efficacy, safety, and pharmacokinetics of the combination of ibrutinib and venetoclax in Japanese patients with R/R MCL.

## Patients and methods

### Study design and patients

M20-075 is an open-label, single-arm, Phase 2 study (NCT04477486). There were 12 sites that participated in the study, of which eight sites enrolled patients. The full eligibility criteria are described in the Supplementary Materials. Briefly, patients aged 20 years or older with pathologically confirmed MCL with at least one measurable disease were enrolled. Patients must have been previously treated with one to five prior lines of therapy including at least one prior rituximab/anti-CD20–containing regimen. Failure to achieve at least a partial response (PR) with, or documented disease progression after, the most recent treatment regimen was required. No prior therapy with ibrutinib or other BTKis was allowed. The full analysis set (FAS) comprised patients who received ≥ 1 dose of study drug. The per protocol set (PPS) excluded FAS patients who were determined to have non-evaluable disease at baseline based on assessment by independent review committee (IRC). The study was approved by institutional review boards and/or independent ethics committees. The study was conducted in accordance with the International Council for Harmonisation guidelines and the Declaration of Helsinki. All patients provided written consent.

### Treatment and assessments

Patients were administered 560 mg ibrutinib once daily as a fixed dose from initial administration with concurrent venetoclax (starting with a ramp-up period to mitigate the risk of TLS). Venetoclax ramp-up consisted of four dose increases every 7 days: patients received a starting dose of 20 mg, then proceeded to 50 mg, 100 mg, 200 mg, and 400 mg venetoclax. DLTs were assessed in the first six patients during the venetoclax ramp-up period for a minimum of 5 weeks and at least 1 week of venetoclax dosing at 400 mg. Further detail pertaining to definition and criteria of DLTs (non-hematologic and hematologic) are described in the supplement. The supplement includes details on ibrutinib and venetoclax dose interruptions and reductions. Patients could continue ibrutinib plus venetoclax for a maximum of 104 weeks followed by ibrutinib monotherapy until disease progression, unacceptable toxicity, or withdrawal of consent. Patients who were high-risk for TLS (at least one lesion > 10 cm or at least one lesion > 5 cm and circulating lymphocytes) and/or baseline creatine clearance < 60 mL/min were followed closely with prophylactic measures including adequate hydration, anti-hyperuricemic agents, labs, and hospitalization [14]. For all ramp-up doses, laboratory values for electrolyte changes suggestive of TLS were reviewed. Laboratory and clinical TLS was assessed per Howard criteria [15]. Minimal residual disease (MRD)-negative remission was defined as undetectable MRD (uMRD) as assessed by flow cytometry (based on a sensitivity of < 0.05% MCL cells per total white blood cells) of bone marrow aspirate and/or peripheral blood from patients who have achieved a CR.

### Endpoints

Clinical response was assessed according to the positron emission tomography criteria of the Lugano classification [16]. The primary endpoint of the study was the rate of CR as best overall response as assessed by IRC [16]. Secondary endpoints were ORR (defined as best overall response of CR or PR as assessed by IRC and investigator); duration of response (DOR; defined as the time from the first occurrence of response to disease progression or death); uMRD rate in patients achieving a CR as assessed by investigator and IRC; CR rate as assessed by investigator; PFS (defined as the time from the date of the first dose of any study drug to the date of investigator-assessed disease progression per Lugano classification or death), OS (defined as the time from the date of the first dose of any study drug to death from any cause), TEAE (treatment-emergent adverse events; defined as any untoward medical occurrence in a patient or clinical investigation patient administered a pharmaceutical product and which does not necessarily have a causal relationship with this treatment) rate and severity according to the National Cancer Institute Common Terminology Criteria for Adverse Events version 5.0, and pharmacokinetic (PK) parameters of ibrutinib and venetoclax. All TEAEs reported from the time of study drug administration until 30 days after discontinuation of study drug.

### Pharmacokinetics

Blood samples were collected on Week 6 Day 1 for the evaluation of venetoclax and ibrutinib PK. Samples were collected pre-dose (0 h) and at 1, 2, 4, 6 and 8 h post-dose. Pharmacokinetic parameters, including peak plasma concentration (C_max_), time to C_max_ (peak time, T_max_), and area under the plasma concentration–time curve over a 24-h dose interval (AUC_24_) were determined using noncompartmental methods. To calculate AUC_24_, venetoclax and ibrutinib concentrations at 24 h were imputed using the 0 h concentrations.

### Statistical analyses

The FAS was used for all efficacy endpoints (except endpoints based on assessment by IRC), safety, PK, and baseline analysis. The PPS was used for endpoints based on assessment by IRC and used as appropriate for investigator assessment endpoints. The estimate and 95% confidence interval (CI) for the CR rate, ORR, and uMRD rate were based on exact binomial distribution. The CR rate was compared to a historical control threshold of 12.5% observed with ibrutinib monotherapy in Japanese patients with R/R MCL using the exact binomial test at a 1-sided overall significance level of 0.025 [10]. For time to event endpoints, survivorship function was estimated by using Kaplan–Meier product-limit method. All analyses were performed using SAS Version 9.4 or later under UNIX operating system.

## Results

### Patient characteristics

A total of 13 patients were enrolled in the FAS, including 12 patients in the PPS. The first patient to initiate therapy was administered a first dose on October 7, 2020, and the last patient to initiate therapy was administered a first dose on November 15, 2021. The data cutoff date was February 9, 2022. Patients in the FAS were predominantly male (77%), and the median age was 71 years (range 59–81; Table 1). Two patients (15%) had an Eastern Cooperative Oncology Group performance status of 1. At baseline, three (23%) patients had bone marrow involvement, three (23%) patients had gastrointestinal involvement, and five patients (38%) had bulky disease ≥ 5 cm. Median number of prior regimens was two (range, 1–3; Table 1).

**Table 1:** Patient demographics and baseline characteristics^a^

Characteristic	PPS (*N* = 12)^b^	FAS (*N* = 13)^c^
Age		
Median (range), years	71 (59–81)	71 (59–81)
Mean (SD), years	69 (6)	70 (6)
≥ 65, *n* (%)	10 (83)	11 (85)
Sex, *n* (%)		
Male,	9 (75)	10 (77)
Female	3 (25)	3 (23)
Relapsed or refractory disease, *n* (%)		
Relapsed	12 (100)	13 (100)
Median no. of prior regimens (range)	N/A	2 (1–3)
Prior regimens, *n* (%)		
1	1 (8)	2 (15)
2	7 (58)	7 (54)
≥ 3	4 (33)	4 (31)
Prior ASCT (%)	3 (25)	3 (23)
ECOG PS, *n* (%)		
0	10 (83)	11 (85)
1	2 (17)	2 (15)
Bone marrow involvement (at baseline), *n* (%)		
Yes	2 (17)	3 (23)
No	10 (83)	10 (77)
GI involvement (at baseline), *n* (%)		
Yes	3 (25)	3 (23)
No	9 (75)	10 (77)
B-symptoms, *n* (%)		
Yes	0	0
No	12 (100)	13(100)
Bulky disease, *n* (%)		
< 5 cm	8 (67)	8 (62)
≥ 5 cm	4 (33)	5 (38)
MIPI score, *n* (%)		
Low	2 (17)	3 (23)
Intermediate	7 (58)	7 (54)
High	3 (25)	3 (23)
Histology, *n* (%)		
Typical	6 (50)	7 (54)
Blastoid	3 (25)	3 (23)
Pleomorphic	2 (17)	2 (15)
Unknown	1 (8)	1 (8)
TLS risk category [14], n (%)		
High	5 (42)	6 (46)
Low	7 (58)	7 (54)

*ASCT* autologous stem cell transplantation, *CR* complete response, *ECOG PS* Eastern Cooperative Oncology Group performance status, *FAS* full analysis set *GI* gastrointestinal, *IRC* independent review committee, *MIPI* Mantle Cell Lymphoma International Prognostic Index, *ORR* overall response rate, *PPS* per protocol set, *TLS* Tumor lysis syndrome

^a^Percentages may add up to more than 100 due to rounding

^b^PPS excludes patients determined to have non-evaluable disease at baseline based on IRC assessment and was used for analysis of IRC-based efficacy endpoints (CR and ORR)

^c^FAS includes all patients who received at least 1 dose of study drug and was used for analyses of non–IRC-based efficacy endpoints, and safety, pharmacokinetic, and baseline analyses

### Efficacy outcomes

**Fig. 1:**
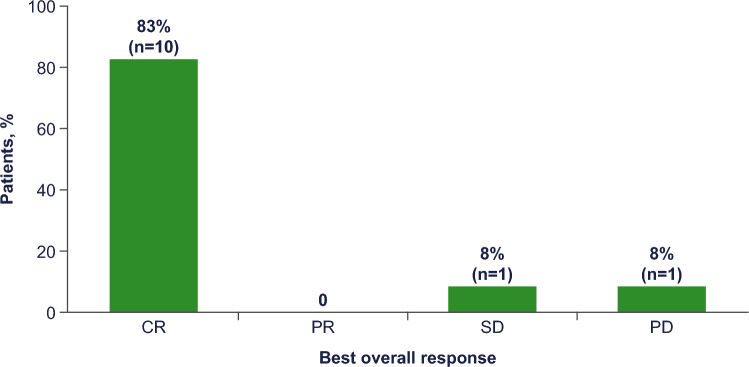
Response rates by IRC (per protocol set). *CR* complete response, *IRC* independent review committee, *PD* progressive disease, *PR* partial response, *SD* stable disease

**Fig. 2:**
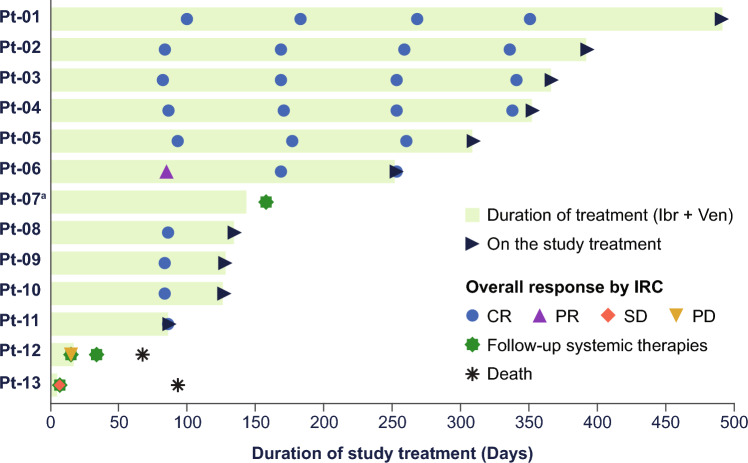
Overall response and duration of treatment. ^a^Pt-07 has no OR by IRC due to having a non-evaluable disease at baseline per IRC baseline assessment. *CR* complete response, *Ibr* ibrutinib, *IRC* independent review committee, *OR* odds ratio, *PD* progressive disease, *PR* partial response, *Pt* patient, *SD* stable disease, *Ven* venetoclax

**Fig. 3:**
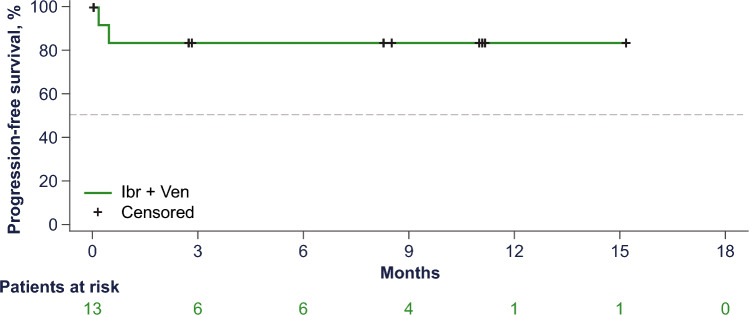
Progression-free survival (full analysis set). *Ibr* ibrutinib, *Ven* venetoclax

**Fig. 4:**
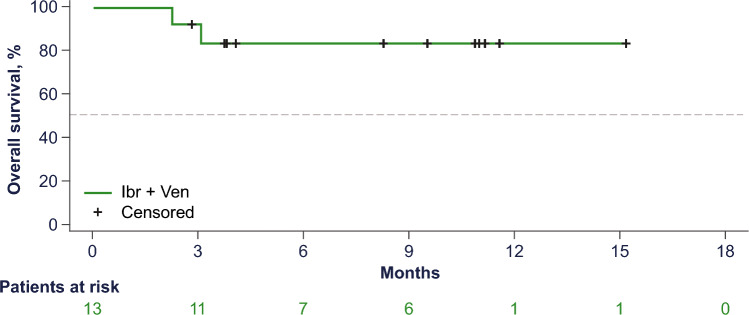
Overall survival (full analysis set)**.** *Ibr* ibrutinib, *Ven* venetoclax

With a median follow-up of 9.6 months, the ORR was 83% (10 of 12 patients in the PPS; 95% CI 51.6–97.9; Fig. 1), with all responding patients achieving a CR as assessed by IRC (best overall response of CR rate: 83%). The primary outcome met statistically significant superiority of venetoclax and ibrutinib against historical reference of ibrutinib monotherapy [10]. The investigator-assessed CR rate was 77% (10 of 13 in the FAS; 95% CI 46.2–95.0), which accounted for all patients who achieved a response. Of the six patients with CR per IRC and investigator assessment that were evaluated for MRD, all six patients achieved uMRD in peripheral blood and/or bone marrow. The median DOR was not reached. The overall response and duration of treatment in the FAS is summarized in the swimmer plot (Fig. 2), with 10 patients still on study treatment, whereas three patients discontinued: two patients due to early PD before reaching 400 mg venetoclax (discontinued on Days 6 and 15), and one patient due to TEAE of squamous cell carcinoma (SCC) of the lung. Nine of 10 patients who responded to treatment achieved CR by the first disease assessment at Week 13, and all patients who responded to treatment (≥ PR) remained in response as of the data cutoff date. The median PFS was not reached (95% CI 0.5–not reached [NR]; Fig. 3), and median OS was not reached (95% CI 3.1–NR; Fig. 4) in the FAS population. The 12-month PFS estimate was 83% (95% CI 48.2–95.6), and the 12-month OS estimate was 84% (95% CI 49.4–95.7).

### Safety outcomes

Patients received study treatment for a median of 20.4 weeks (range, 0.9–70.1). All 13 patients in the FAS experienced ≥ 1 TEAE, and 31% (4 of 13 patients) experienced a TEAE of Grade ≥ 3. The most common any-grade TEAEs that occurred in ≥ 20% of patients included diarrhea (46%), neutropenia (31%), hyperkalemia (23%), leukopenia (23%), skin infection (23%), nausea (23%), and thrombocytopenia (23%; Table 2). The most common Grade ≥ 3 TEAEs that occurred in ≥ 10% of patients (≥ 2 patients) was neutropenia (23%; Table 2). Two patients (15%) had serious TEAEs: one patient had Grade 3 hemorrhoids and Grade 4 sepsis, and the other had Grade 4 neutropenia and Grade 3 SCC of the lung (Table 2). Neutropenia requiring granulocyte colony-stimulating factor (G-CSF) treatment and sepsis were each assessed by the investigator to be related to both venetoclax and ibrutinib. No DLTs or cases of laboratory or clinical TLS were observed.

**Table 2:** Most common treatment-emergent adverse events

TEAE, *n* (%)^a^	All grades	Grade ≥ 3	Serious
Diarrhea	6 (46)	0	0
Neutropenia	4 (31)	3 (23)	1 (8)
Thrombocytopenia	3 (23)	0	0
Leukopenia	3 (23)	0	0
Hyperkalemia^b^	3 (23)	0	0
Skin infection	3 (23)	0	0
Nausea	3 (23)	0	0
Hypertension	2 (15)	1 (8)	0
Hypokalemia	1 (8)	1 (8)	0
Hemorrhoids	1 (8)	1 (8)	1 (8)
Sepsis	1 (8)	1 (8)	1 (8)
Squamous cell carcinoma of lung	1 (8)	1 (8)	1 (8)

^a^Includes TEAEs of any grade reported in ≥ 20% of patients, grade ≥ 3 TEAEs reported in ≥ 5% of patients, and/or any-grade serious TEAE reported in any patient. Two patients had two incidences of SAEs

^b^Not related with TLS by the investigators and did not meet Howard’s criteria

*SAE* serious adverse event, *TEAE* treatment-emergent adverse event, *TLS* tumor lysis syndrome

TEAEs led to venetoclax interruptions in 38% of patients (5 of 13 patients), dose reduction in 31% (4 of 13 patients), and discontinuation in 8% (1 of 13 patients; Table 3). TEAEs led to ibrutinib interruptions in 38% of patients (5 of 13 patients), dose reduction in 15% (2 of 13 patients), and discontinuation in 8% (1 of 13 patients; Table 3). One patient discontinued both ibrutinib and venetoclax due to a Grade 3 SCC of the lung, which was deemed unrelated to either study drug. One case of serious neutropenia (absolute neutrophil count > 1500) required treatment with G-CSF and resolved. No deaths related to TEAEs occurred. Two patients died > 30 days after the last treatment dose due to progressive disease.

**Table 3:** Ibrutinib and venetoclax dose modifications due to adverse events in the full analysis set

	FAS (*N* = 13)	
	Ibrutinib	Venetoclax
TEAE leading to discontinuation, *n* (%)	1 (8)	1 (8)
Squamous cell carcinoma	1 (8)	1 (8)
TEAE leading to reduction, *n* (%)	2 (15)	4 (31)
Atrial fibrillation	1 (8)	0
Sepsis	1 (8)	1 (8)
Nausea	0	1 (8)
Neutropenia	0	2 (15)
TEAE leading to interruption, *n* (%)^a^	5 (38)	5 (38)
Allergic reaction	1 (8)	1 (8)
C-reactive protein increased	1 (8)	1 (8)
Diarrhea	1 (8)	1 (8)
Erythema multiforme	1 (8)	1 (8)
Hemorrhoid	1 (8)	1 (8)
Hyperkalemia	1 (8)	1 (8)
Neutropenia	1 (8)	1 (8)
Pyrexia	1 (8)	1 (8)
Sepsis	1 (8)	1 (8)
Squamous cell carcinoma	0	1 (8)

*FAS* full analysis set, *TEAE* treatment-emergent adverse event

^a^Some patients experienced multiple events

### Pharmacokinetic outcomes

Pharmacokinetic data were available from 11 patients. Two patients discontinued treatment prior to the PK sampling day (Week 6 Day 1), and no PK data were available for those patients. Steady-state plasma concentration versus time profiles for venetoclax and ibrutinib are provided in the data supplement (Supplemental Fig. 1). Peak venetoclax and ibrutinib concentrations were observed at 8 h and 4 h, respectively. A summary of the steady-state pharmacokinetic parameters of venetoclax and ibrutinib are presented in Supplemental Table 1 and Supplemental Fig. 1.

## Discussion

The primary efficacy outcome of CR rate (83%) in this study shows statistically significant superiority of venetoclax and ibrutinib compared to ibrutinib monotherapy (historical CR rate of 12.5%) in Japanese patients with R/R MCL [10]. This compares favorably to the CR rate of 71% (positron emission tomography confirmed) observed in the AIM study after a median 16 months of follow-up and the CR rate of 62% observed in the SYMPATICO study after a median 31 months of follow-up [13, 14]. Additionally, all six MRD-evaluable patients with a CR also achieved uMRD and were still on treatment as of the data cutoff date. All 10 PPS patients who completed the venetoclax ramp-up and reached venetoclax 400 mg achieved a best response of CR despite the fact that many of the patients enrolled had high-risk disease features, including an intermediate or high Mantle Cell Lymphoma International Prognostic Index score (10 of 12 in PPS; 83%), disease with blastoid (3 of 12 in PPS; 25%) or pleomorphic (2 of 12 in PPS; 17%) morphology, and bulky disease ≥ 5 cm (4 of 12 in PPS; 33%) at baseline.

Prior studies have demonstrated the efficacy of ibrutinib and venetoclax as single-agent monotherapy [7, 11, 17]. The mechanistic rationale for combining these two agents was derived from the synergistic effect of concurrent BTK inhibition and BCL-2 inhibition described in preclinical studies. Exposure of leukemic patient cells to the combination of ibrutinib and venetoclax for 72 h increased induction of apoptosis compared to each agent alone [18]. A separate study using MCL cell lines confirmed the synergistic effect of ibrutinib and venetoclax on proliferation inhibition and apoptosis through perturbation of the BTK, AKT, and BCL-2 pathways [19]. The therapeutic synergy of this combination is predicted to be a result of not only the different critical pathways of each agent but also the enhanced targeting effect of each agent on the other.

Venetoclax exposures in combination with ibrutinib in this patient cohort was approximately 2.5-fold higher compared to exposures observed in Japanese patients with chronic lymphocytic leukemia receiving venetoclax monotherapy [20]. This is consistent with previously reported increases in venetoclax exposures upon co-administration with ibrutinib [21]. Venetoclax did not appear to influence ibrutinib exposures, as ibrutinib exposures observed in combination with venetoclax in Japanese patients within this study were comparable to exposures reported in Japanese patients receiving ibrutinib 420 mg monotherapy [22]. Additionally, the possibility of venetoclax impacting ibrutinib PK has been excluded by a within-subjects comparison in the CAPTIVATE study [21]. Ibrutinib and venetoclax combination therapy was well-tolerated, with one patient discontinuing treatment due to SCC of the lung unrelated to study treatment. Serious TEAEs were observed in two patients. Common TEAEs observed in this study were largely consistent with adverse events reported in prior studies of ibrutinib and venetoclax monotherapy [7–11, 23].

Due to its potent anti-apoptotic effect, treatment with venetoclax carries risk of TLS [24]. Interestingly, previous investigations have described the influence of ibrutinib lead-in on reducing lymph node bulk and absolute lymphocyte count, reducing the risk of TLS [25]. A retrospective analysis of 20 patients with R/R MCL and prior BTKi exposure who were treated with venetoclax monotherapy (doses ranging from 20 to100 mg) reported no clinical cases of TLS with the drug being well tolerated [26]. In the AIM study where 560 mg ibrutinib was administered for 4 weeks prior to venetoclax ramp-up starting at 50 mg daily, TLS events were reported in two patients with high tumor burden; this led to a revision of the protocol to reduce the venetoclax starting dose from 50 to 20 mg per day [13]. Subsequently, seven additional patients were treated using the revised schedule, and no cases of TLS occurred. In the safety run-in cohort of the SYMPATICO study of 21 patients with R/R MCL who were concurrently treated with 560 mg ibrutinib and 400 mg venetoclax (ramping up from an initial dose of 20 mg), no instances of clinical TLS and one occurrence of laboratory TLS (in a patient at increased risk for TLS) were reported [14]. Although six of 13 patients in the current study were at high risk for TLS at baseline per established TLS risk categories [14], no TLS events were observed among 13 patients with concurrent treatment of ibrutinib and venetoclax ramp-up using an initiation dose of 20 mg once daily. Of note, three cases of hyperkalemia were observed, but none one them were associated with TLS per investigators, nor did they meet Howard’s criteria [15]. Concurrent initiation with ibrutinib and venetoclax is desirable and manageable considering the low risk of TLS events after a 5-week venetoclax ramp-up from 20 mg.

Despite the limitations of this study, including the small sample size and lack of a control group, the combination of ibrutinib and venetoclax demonstrated a clinically meaningful benefit in Japanese patients with R/R MCL. High CR rates and a tolerable safety profile with ibrutinib plus venetoclax were observed in elderly patients, representing a real-world population. The findings of this Phase 2 study reinforce the continued evaluation of the combination of ibrutinib and venetoclax, including the ongoing Phase 3 SYMPATICO study, for the treatment of R/R MCL.

### Supplementary Information

Below is the link to the electronic supplementary material.

Supplementary file1 (EPS 659 KB)

Supplementary file2 (PDF 267 KB)
