# Supplementary figures and images for: Phase 2 study of ibrutinib plus venetoclax in Japanese patients with relapsed/refractory mantle cell lymphoma
Source: Int J Clin Oncol. 2023 Dec 29;29(2):232–40. doi: 10.1007/s10147-023-02443-6 (PMC10808627; doi:10.1007/s10147-023-02443-6)

**a**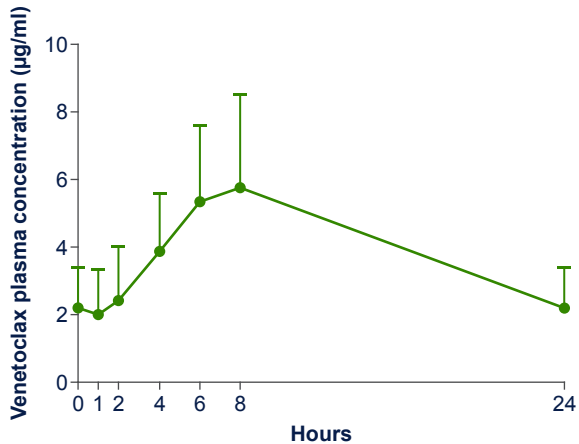**b**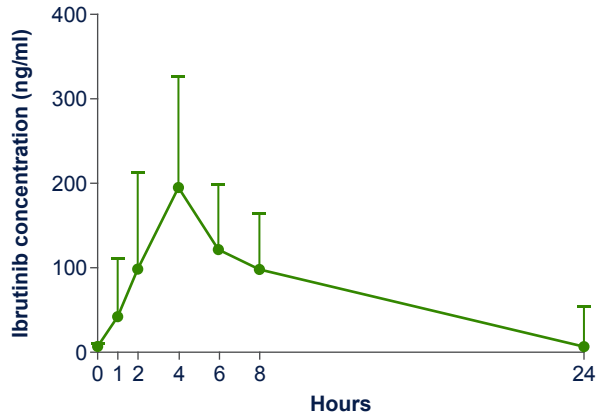

Supplement: Supplementary file 2 — Supplementary file2 (PDF 267 KB) [file 10147_2023_2443_MOESM2_ESM.pdf]
